# Suppression of the CD8 T cell response by human papillomavirus type 16 E7 occurs in Langerhans cell-depleted mice

**DOI:** 10.1038/srep34789

**Published:** 2016-10-06

**Authors:** K. Jemon, C.-M. Leong, K. Ly, S. L. Young, A. D. McLellan, M. H. Hibma

**Affiliations:** 1 Department of Microbiology and Immunology, Otago School of Medical Sciences, University of Otago, Dunedin, New Zealand; 2 Department of Pathology, Dunedin School of Medicine, University of Otago, P O Box 56, Dunedin, New Zealand

## Abstract

Human papillomavirus (HPV) is an epitheliotropic virus that is the primary causal agent for cervical cancer. Langerhans cells (LC) are skin antigen presenting cells that are reduced in number in HPV-infected skin. The aim of this study was to understand the immune-modulatory effects of HPV16 E7 on LC and on the CD8 T cell response to a skin-expressed antigen. To test this, HPV16 E7 was expressed in mouse skin keratinocytes with the model antigen ovalbumin (Ova). Similar to what is observed in HPV-infected human skin, LC numbers were significantly reduced in E7-expressing mouse skin. This shows that expression of the E7 protein alone is sufficient to mediate LC depletion. Expression of E7 with Ova in keratinocytes strongly suppressed the Ova-specific CD8^+^ T cell response in the skin draining lymph node. When tested in LC-ablated mice, the CD8 T cell response to skin-expressed Ova in control mice was not affected, nor was the T cell response to Ova restored in E7-expressing skin. These data indicate a role for E7 in regulation of LC homeostasis in the skin and in suppression of antigen specific CD8 T cell expansion, but suggest that these two effects occur independent of each other.

Human papillomavirus (HPV) is an epitheliotropic virus that is the primary etiological agent of cervical cancer^1^,^2^. The high-risk genotypes 16 and 18 are most prevalent worldwide and are detectable in more than 75% of all cervical tumours^3^. It has been established that continuous expression of the E6 and E7 oncoproteins is necessary to maintain a transformed phenotype during cervical carcinogenesis^1^. There is an increasing body of evidence that E6 and E7 also contribute to HPV evasion of the host immune response^4^.

HPV infections are very common, especially among sexually active individuals. It is estimated that 50 to 80% of sexually active men and women acquire HPV infections throughout their lives^5^. Although the prevalence of HPV is high, the majority of infections do eventually resolve, generally within 2 years. Around 10–20% of the HPV-infected individuals fail to clear the virus effectively and remain HPV DNA positive. Individuals with persistent infections with high-risk types have a much greater chance of progression to high-grade CIN and invasive carcinoma^6^,^7^.

Lesion regression is associated with activation of an adaptive immune response to HPV, with CD8 and CD4 T cells likely being the major effector cells mediating the response^8^. CD8 T cell activation is contingent on presentation of viral antigens by professional antigen presenting cells (APC) and typically is dependent on three signals: APC presentation of peptide with MHCI to the T-cell receptor on the T cell, interaction between co-stimulatory molecules on the APC with respective ligands on the T cell, and inflammatory cytokine secretion (including IL-12) by the APC^9^.

Persistence of viral infection is primarily attributed to the absence of an effective immune response that is likely contributed to by poor presentation of viral antigens. HPV is non-cytolytic and infection is restricted to keratinocytes (KC), both factors that may limit the availability of antigen for presentation to T cells. The professional APCs resident at the site of HPV infection are Langerhans cells (LC), which, because of their location, are considered likely to be important for immune modulation of HPV infection and HPV-induced lesions. However their role in presentation of HPV antigens is challenging to test directly in the immunologically well-defined mouse system, as HPV has a strict tropism to humans.

There is evidence supporting HPV interference of antigen presentation. Langerhans cell homeostasis is regulated in HPV infected lesions, resulting in a net loss of LC at the site of infection^10^. HPV also interferes with antigen presentation and processing machinery^11^,^12^, and alters chemokine and cytokine expression by LC^13^,^14^.

The purpose of this study is to determine if expression of HPV16 E7 in basal and suprabasal keratinocytes is sufficient to regulate LC homeostasis and function *in vivo*, and the CD8 T cell response to a skin-expressed antigen. We show that, comparable to human infected skin, LC numbers are reduced in E7-expressing mouse skin. In addition, co-stimulatory molecule expression on LC from E7-expressing skin is increased. We found that HPV16 E7 strongly suppresses antigen specific T cell proliferation to co-expressed ovalbumin (Ova). Perhaps surprisingly, depletion of LC from control or E7 expressing skin did not alter the magnitude of the T cell response. This suggests that LC are not essential for activation of the T cell response, nor are they required for E7-mediated suppression of the T cell response to skin-expressed antigen.

## Results

### The frequency of LC is decreased in the ear of mice transduced with HPV16 E7

We wished to determine if expression of E7 is sufficient to mediate the reduction in LC observed in human HPV-infected skin. To test this E7, or E7 cloned in reverse, and luciferase, regulated by the same K14 promoter region, were expressed in Lang-DTR transgenic mouse (without DT treatment) ear skin *in vivo*. Expression of the transduced genes over time was determined by measuring the bioluminescence signal emitted by the luciferase reporter, following injection of luciferin. Comparable levels of luciferase expression were observed up to day 14 (Supplementary Fig. 1).

**Figure 1:**
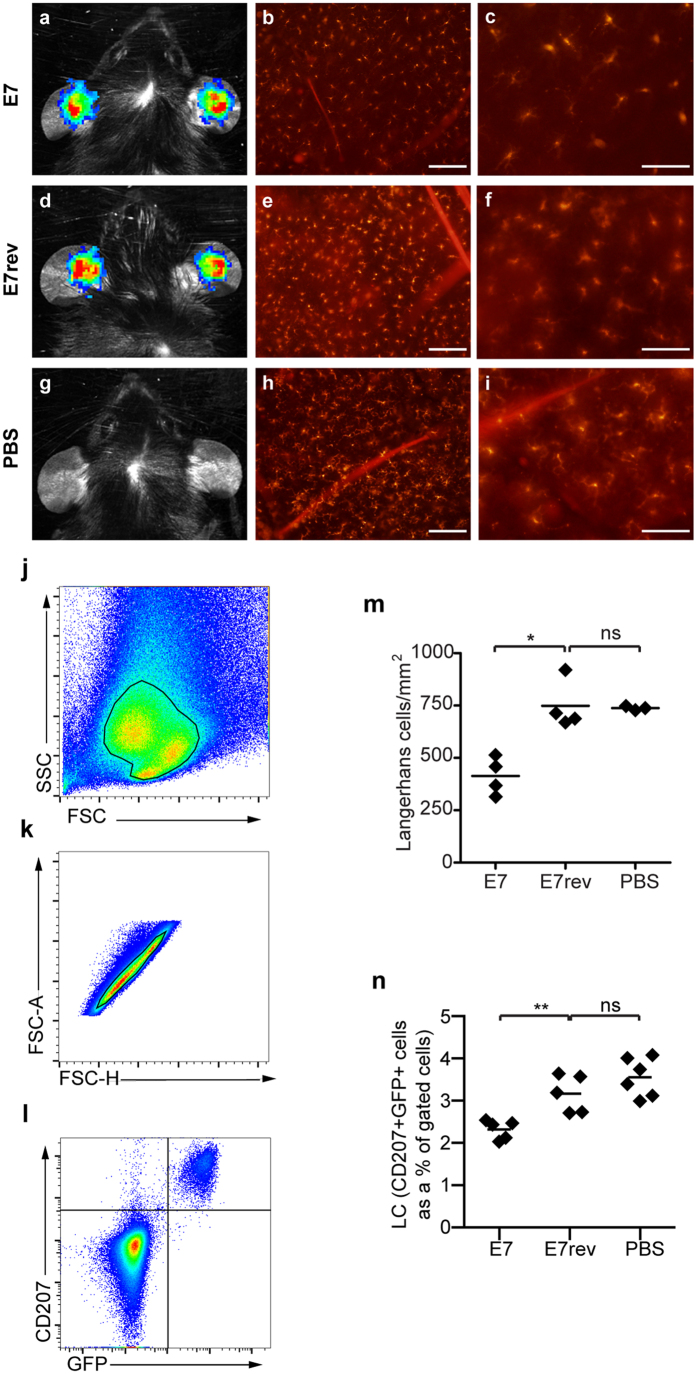
The number of LC is reduced in HPV16 E7 expressing epidermis. Lang-DTR mice were injected intradermally beneath the ear skin with either 1 × 10^7^ K14 E7 Luc/Ova or K14 E7rev Luc/Ova lentivirus particles, or treated with PBS. Ten days following transduction, *in vivo* bioluminescence imaging was carried out to measure luciferase gene expression. Representative images show comparable bioluminescence signal from K14 E7 Luc/Ova (**a**) and K14 E7rev Luc/Ova (**d**) expressing keratinocytes in both groups and no signal from the PBS control group (**g**). Epidermal sheets were prepared from the transduced mouse ear skin and stained with anti-CD207-conjugated Alexa-546, to visualize LC. Red stained LC within the transduced area of K14 E7 Luc/Ova (**b**), K14 E7rev Luc/Ova (**e**) and PBS control groups (**h**) are shown and enlarged regions are depicted in (**c**), (**f**) and (**i**) respectively. Scale bars, 50 μm. Individual epidermal LC numbers are shown graphically (**m**), with the median depicted for each group (*n* = 3–4 per group); *P < 0.05 (Mann-Whitney U). LC numbers in ear skin were confirmed using flow cytometry. The gating strategy used to determine the percentage of LCs from ear cell suspensions is shown. Single cell suspensions of epidermal cells were distinguished using forward and side scatter (**j**), a gate applied to single cells (**k**) and double positive CD207^+^ and GFP^+^ LC were gated (**l**). The percentage of LC in the gated population is shown graphically (**n**), as is the mean for each group (n = 5/6 per group); *P < 0.05 **P < 0.01 (Mann-Whitney U).

Epidermal sheets were harvested from E7 expressing epidermis and control skin transduced with E7 cloned in reverse at day 10, and stained with an antibody specific for langerin/CD207. Expression of the transduced genes at day 10 was confirmed by measuring the bioluminescence signal (Fig. 1a,d,g). Consistent with what has been observed in human skin infected with HPV16, there was a significant decrease (*P* < 0.05, Mann-Whitney U) in the number of LC in HPV16 E7 expressing skin when compared with luciferase expressing control epidermis (Fig. 1b,c,e,f,h,i and m). To ensure that expression of luciferase alone did not affect LC number, luciferase-expressing ear skin was compared to PBS control skin and LC density was determined to be comparable (Fig. 1m).

To further confirm those data, single-cell suspensions of ear skin cells from Lang-DTR mouse skin (without DT treatment) expressing E7 and luciferase, E7rev and luciferase, or a PBS injected control were prepared, cells stained with anti-CD207 and the LC enumerated using flow cytometry. The population of cells was gated using forward and side scatter, and a single cell gate applied. LCs were additionally identified by green fluorescent protein (GFP) positivity (Fig. 1k,l), as the Lang-DTR mice express GFP regulated by the langerin promoter^15^. In agreement with the results from the immunohistochemistry analysis, there were significantly fewer (*P* < 0.05, M-W U) CD207^+^GFP^+^ LC in E7 and luciferase-expressing epidermis compared to luciferase expressing control skin or PBS injected skin (Fig. 1n). Taken together, these results clearly demonstrate that expression of HPV16 E7 in KC is sufficient to cause a significant reduction in the number of LC resident in the epidermis.

### Expression of co-stimulatory molecules on LC from HPV16 E7-transduced skin

**Figure 2:**
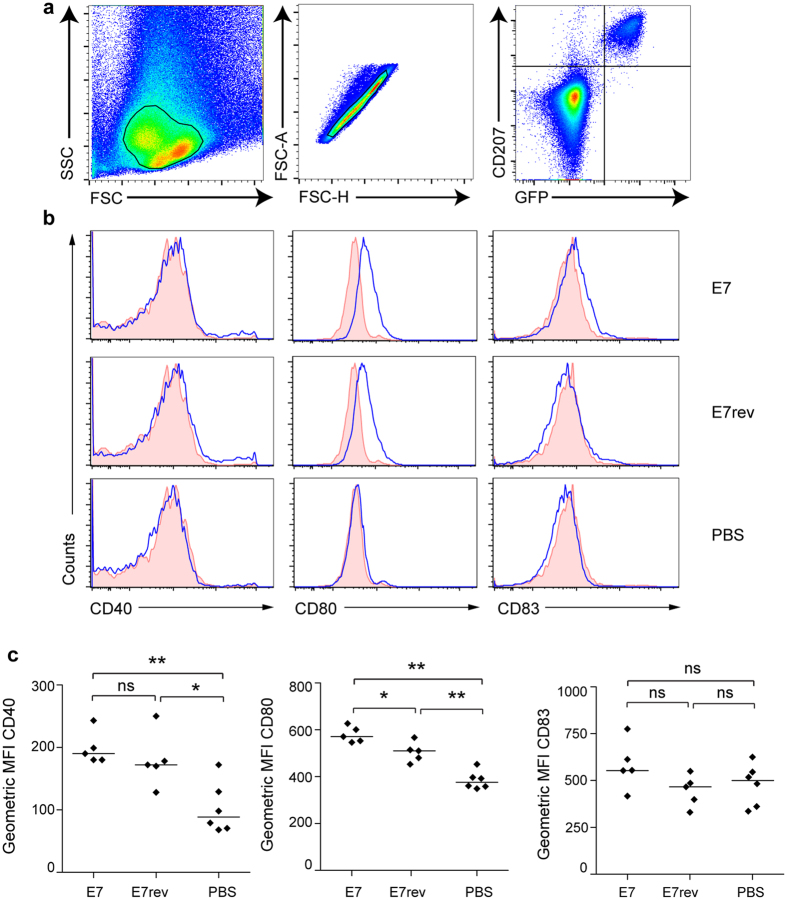
Co-stimulatory marker expression on LC from K14 E7 expressing mouse skin is increased. Lang-DTR mice were injected i.d. beneath the ear skin with 5 × 10^6^ K14 E7 Luc or K14 E7rev Luc lentivirus particles, or with PBS. Ten days following transduction, epidermal ear suspensions were prepared and analyzed by flow cytometry. (**a**) The gating strategy applied to identify the single, CD207, GFP positive cells that were analysed for co-stimulatory molecule expression. (**b**) Surface expression of CD40, CD80, and CD83 on LC was analyzed. Representative histograms are shown for a minimum of 5 mice in each group. The shaded histograms correspond to isotype control staining. (**c**) The geometric mean fluorescence intensity (MFI) of CD40, CD80, and CD83. Lines show the median for at least 5 mice per group. *P < 0.05; **P < 0.01 (Mann-Whitney U).

The reduction of the quantity of LC in the E7-transduced mice prompted us to investigate the activation status of the remaining skin-resident LC. LC that reside in the skin are immature; they typically express low levels of the co-stimulatory molecules CD40, CD80, CD83 and CD86, and have the capacity to take up antigen and process antigen. On activation, LC migrate from the epidermis to the lymph node, undergoing maturation, and expressing increased levels of MHCII and co-stimulatory markers^16^,^17^. To test if the activation state of the skin-resident LC is altered when E7 is expressed in the epidermis, surface expression of CD40, CD80 and CD83 on the LC purified from K14 E7 Luc/Ova and K14 E7rev Luc/Ova transduced skin was measured. Overall, there was a modest increase in expression of CD80 (*P* < 0.05, M-W U) on LC harvested from the HPV16 E7 expressing epidermis when compared with skin transduced with the E7 gene in the reverse orientation (Fig. 2), but no change in CD40 or CD83. We note that the LC from ear skin transduced with lentiviral vectors also had a modest increase in CD40 and CD80 expression (*P* < 0.005: M-W U) relative to ears that had been injected with PBS. Overall, co-stimulatory molecule expression was not at a level consistent with the multiple-fold increases typically seen on activated LCs from the lymph nodes, indicating only limited activation of LC in E7-transduced skin.

### HPV16 E7 suppresses CD8^+^ T cell response against expressed ovalbumin

**Figure 3:**
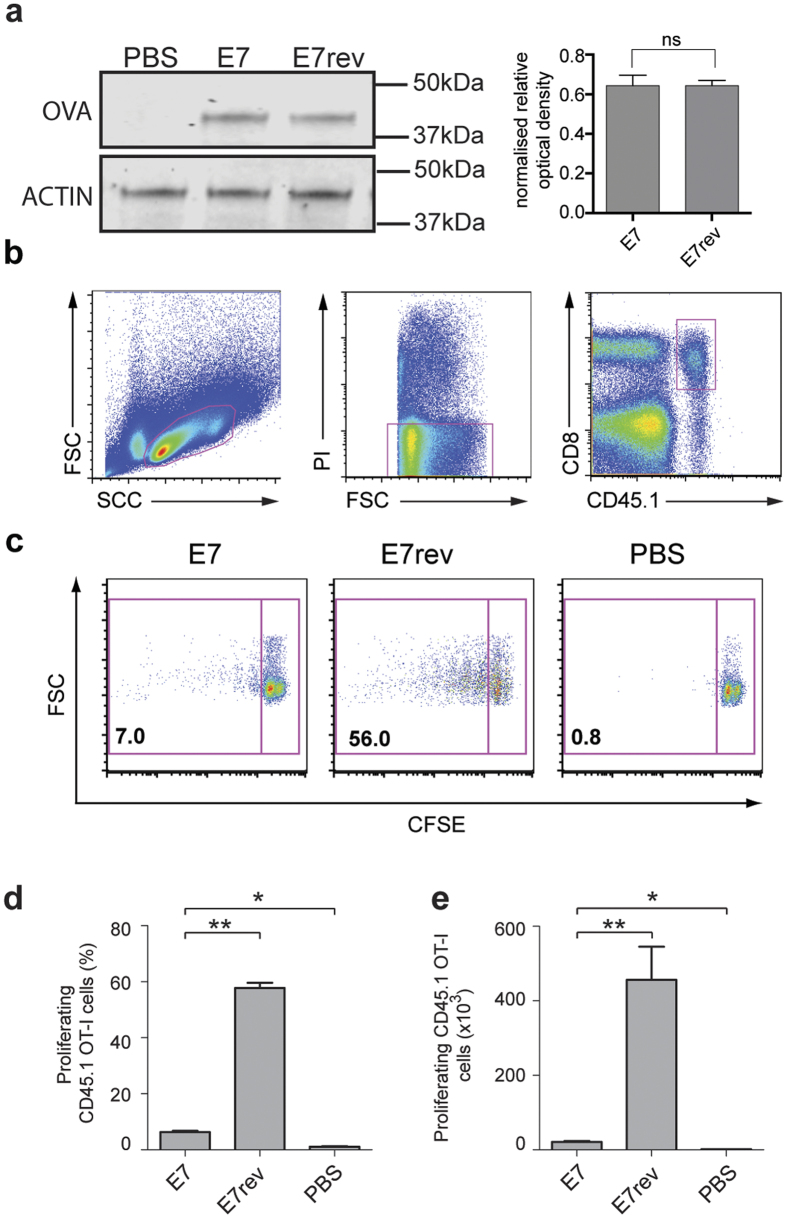
The *in vivo* T cell proliferative response to Ova in the draining lymph node is reduced in mice expressing K14 E7 in the epidermis. Lang-DTR mice were injected i.d. beneath the ear skin with 1 × 10^7^ K14 E7 Luc/Ova, or K14 E7rev Luc/Ova lentivirus particles, or PBS. Seven days post transduction, 4 × 10^6^ CFSE-labelled CD45.1 OT-I cells were adoptively transferred into each mouse intravenously. Five days after transfer, ear skin was harvested and expression of the Ova in the epidermis, and the actin loading control, was determined by western blot. Densitometry was carried out to quantify the amount of protein for each of the bands. The density relative to the loading control is shown for triplicate samples (**a**). The cervical draining lymph nodes were recovered, and analysed by flow cytometry to determine the proliferation of the CFSE-labelled CD45.1, CD8^+^ OT-I T cells. The gating strategy used to identify those cells is shown in (**b**). Representative dot plots of proliferating CFSE-labelled OT-1 T cells are shown for each group (**c**). The percentage (mean ± SEM) (**d**) and absolute number (mean ± SEM) (**e**) of CD45.1 OT-I proliferating cells are shown. *n* = 5; *P < 0.05, **P < 0.005 (Mann-Whitney U).

To test the hypothesis that E7 expression results in impaired T cell responses to co-expressed antigen, we directly measured the OT-I CD8^+^ T cell proliferative response against Ova co-expressed with HPV16 E7 transduced epidermal KC. Firstly we confirmed that comparable amounts of Ova were expressed in equivalent numbers of skin cells transduced with either the K14 E7 Luc/Ova or the K14 E7rev Luc/Ova lentivirus by western blot (Fig. 3a). We then measured the proliferative response to skin expressed Ova. Evidence of *in vivo* proliferation of the OT-I T cells was readily detected in response to Ova expressed in non-DT treated LangDTR mice transduced with K14 E7rev Luc/Ova (Fig. 3b–d). In contrast, when E7 was co-expressed with Luc/Ova, there was a pronounced and significant decrease (*P* < 0.01; M-W U test) in Ova-specific CD8^+^ T cell proliferation. From these data we concluded that expression of E7 in basal KC impaired the CD8 T cell response to a co-expressed antigen.

### HPV16 E7 down-regulation of the CD8^+^ T cell response can occur independently of LC

Evidence supports LC priming of a CD8^+^ T cell response *in vitro*^18^,^19^ and *in vivo*^20^. However the role of LCs in activation of an immune response has been a source of debate, with some workers maintaining that these antigen-presenting cells are predominantly tolerogenic^21^. We therefore wished to establish if LCs contribute to activation of the CD8 T cell response to Ova expressed in KC, and to determine if LCs contribute to the suppression of the Ova response that we observed when E7 was co-expressed with Ova in KC.

**Figure 4:**
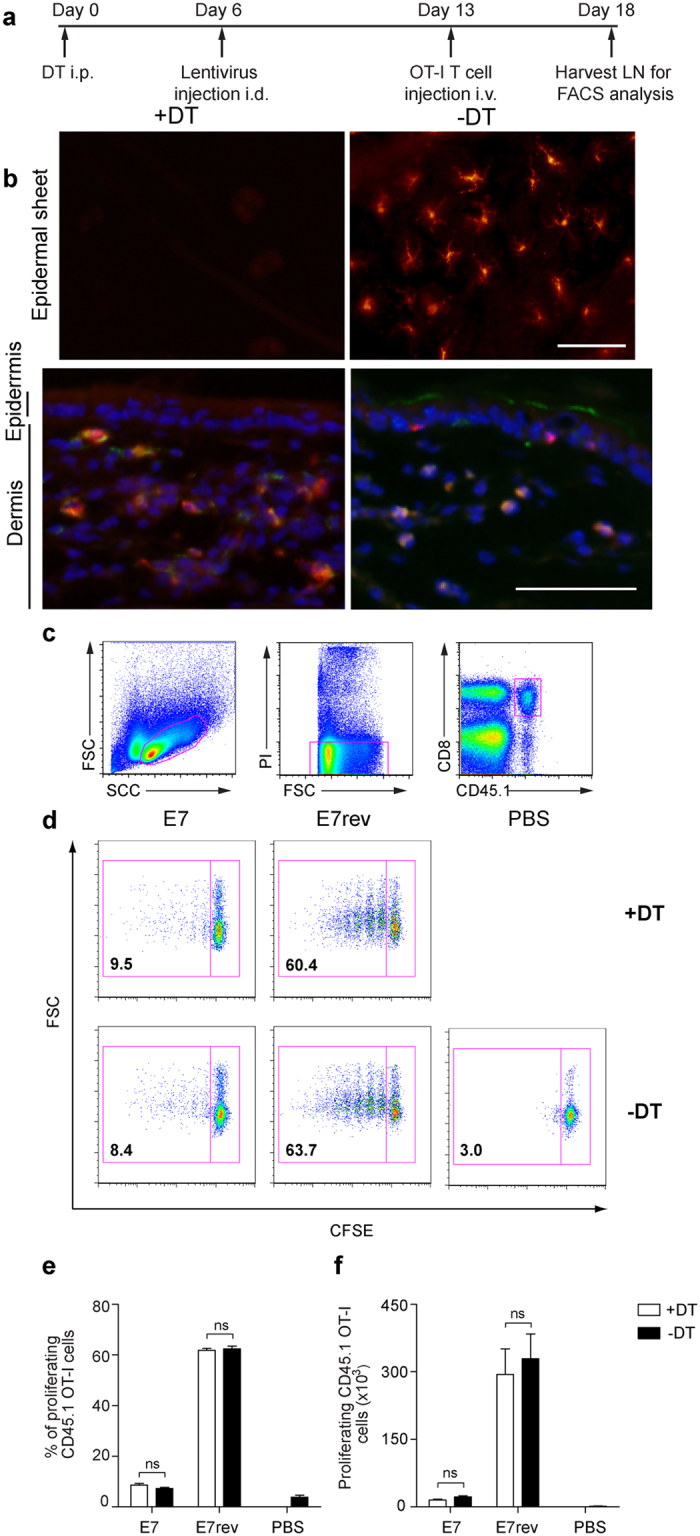
Suppression of the T cell *in vivo* proliferative response to Ova co-expressed with E7 in the skin of the Lang-DTR mouse is not restored when LC are depleted. The experimental outline is shown schematically (**a**). To confirm depletion LCs and retention of CD103^+^, Langerin^+^ DCs following DT treatment, epidermal sheets stained for CD207 (red), and tissue sections were stained with CD103 (green), CD207 (red), and DAPI. (**b**); Scale bars, 50 μm. Cervical-draining lymph nodes were recovered five days after CFSE-labelled OT-1 cells were adoptively transferred into mice. Single cell suspensions were analysed by flow cytometry for CFSE-labelled CD45.1 OT-I T-cell proliferation. The gating strategy used to identify CD45.1^+^, CD8^+^ single cells is shown (**c**). Representative dot plots are shown for each group (**d**). The percentage (mean ± SEM) (**e**) and absolute numbers (mean ± SEM) (**e**) of CD45.1 OT-I proliferating cells are shown. *n* = 5; ns, not significant (Mann-Whitney U).

To address this, we administered DT to the Lang-DTR transgenic mouse, to selectively deplete langerin positive cells^15^,^22^,^23^. At the 1 μg DT dose used here, rapid depletion of LC and langerin positive DCs occurs. The DC subsets are gradually restored after day 5, whereas the LCs are not fully restored until 6 weeks after treatment^15^. Lang-DTR mice (5 mice/group) were treated with DT 6 days prior to lentivirus transduction or left untreated, as outlined in the Fig. 4a. Immunofluorescence staining of ear skin (sheets and skin sections) for langerin positive cells was carried out at day 6 post-DT treatment to determine if LCs and/or dermal DC had repopulated the ear skin. We readily found CD207^+^CD103^+^ dermal dendritic cells at day 6, but the epidermis remained devoid of the CD207^+^ langerin positive cells at that time (Fig. 4b). We also examined sectioned ear skin at day 18 (Supplementary Fig. 2) and similarly did not detect LC in the epidermis, but could detect langerin positive cells in the dermis at that time.

DT treated LangDTR mice were transduced either with K14 E7 Luc/Ova or K14 E7rev Luc/Ova lentivirus, or injected with PBS only. Seven days post-transduction, when LC remained depleted but langerin^+^ dermal DC restored, CFSE-labelled CD45.1 OT-I cells were adoptively transferred into the mice. Cervical lymph nodes were harvested 5 days later and pooled, and the proliferation of the transferred live, CD8+ CFSE-labelled CD45.1 OT-I cells (Fig. 4c) was measured.

Consistent with our other data, there was robust proliferation of OT-I transgenic cells from the ear-draining lymph nodes of mice transduced with K14 E7rev Luc/Ova (Fig. 4d,e). LCs did not appear to be necessary for the CD8 T cell response to Ova, as depletion of LCs prior to transduction did not alter the magnitude of the proliferative response. Similarly, these experiments confirmed our data showing that there was a significant decrease in Ova-specific CD8^+^ T cell proliferation in mice transduced with K14 E7 Luc/Ova. Again, LC depletion did not significantly alter, and did not restore, the proliferative response to that of the E7rev lentiviral transduced skin. These results indicate that LC depletion did not alter the CD8^+^ T cell response to Ova expressed in skin KC and furthermore did not restore the CD8^+^ T cell response in mice transduced with E7 and Ova.

## Discussion

It has previously been reported that LC numbers are reduced in HPV16 infected skin, which was proposed to contribute to evasion of immunity by the virus^24^,^25^,^26^. Here we show that expression of E7 oncoprotein of HPV16 results in reduced LC density in the epidermis, activation of residual LC, and suppression of draining lymph node T cell proliferation to co-expressed antigen in the skin. Importantly, our data show that expression of HPV16 E7, in the absence of other HPV16 proteins, is sufficient to cause these effects.

Expression of the adhesion molecule E-cadherin on both KC and LC is required for retention of LC within the epithelium^27^,^28^. Expression of E-cadherin on the basal and suprabasal KC in HPV-infected biopsy specimens is significantly decreased when compared to normal tissue and directly correlates with reduced LC number^25^,^29^. E-cadherin expression is down-regulated on HPV16 E7^30^,^31^ or E6^25^ expressing human and mouse (data not shown) KC *in vitro*, and co-expression of these proteins further reduces E-cadherin expression^32^. E7-induced down-regulation of E-cadherin may contribute to the reduced LC density we observe in this study.

We have previously examined LC density in the K14 E7 transgenic mouse and found that there was an overall increase in the number of LC in K14 E7 mouse skin, and that the LCs were at least partially activated^33^. We propose that LC density and activation status differs between these two models as a result of secondary effects of the skin microenvironment that are consequential of E7 expression. Long-term expression of E7 in the K14 E7 transgenic mouse causes skin hyperplasia and chronic inflammation. These characteristics are shared by high-grade HPV lesions in humans^34^, in which increased numbers of LC are typically found. In contrast, there is no evidence of inflammation or hyperplasia when E7 is expressed in the lentivirus model. Chronically inflamed mouse skin is populated by short-term LCs that differentiate from infiltrating Gr-1^+^ macrophages^35^, which would account for the overall increase in LC number in the skin that was observed in the K14 E7 transgenic mouse. In contrast, the overall reduction in LCs in the E7 transduced skin is suggestive of the loss of the long-term LCs. The difference in LC regulation in the two models therefore could be accounted for if long-term but not short-term LC are regulated by E7 expression in the skin.

LCs in the skin maintain skin-resident regulatory T cells (Treg)^36^, mediating tolerance. Treg maintenance by LCs is dependent on MHCII and is associated with somewhat increased expression of CD86 but no change in CD80 or CD83^37^. Although we did not measure CD86 in this study, CD86 is increased on LCs in the K14 E7 mouse, and peripheral CD8 T cell suppression is mediated by Tregs in that model^38^. In the human, residual LCs can be found in patches in HPV6/11-infected tissue and frequently co-localise with Treg^24^, suggestive of a role for LCs in Treg maintenance.

Here we show that E7 expression impairs the CD8 T cell response. The almost complete ablation of CD8 T cell proliferation to antigen co-expressed with E7 in epidermal KC is consistent with T cell suppression observed in the K14 E7 transgenic mouse. In that model the suppression is sufficient to prevent rejection K14 E7 mouse skin when transplanted onto a syngeneic immune competent mouse^39^,^40^. Several regulatory effects of E7 may contribute to the ablation of the T cell response to antigen co-expressed with E7 in the skin. CD8 T cell activation requires peptide to be presented with MHC I, which engages with the T cell receptor. HPV16 E7 represses the MHC I promoter, and reduces transporters of antigenic peptides (TAP) 1 and low molecular weight protein (LMP) 2 promoter activity to a lesser degree^41^. Reduced expression of MHC I^12^,^42^, TAP1 and TAP2, and proteosome subunits LMP2 and LMP7 has been confirmed in lesions from patients with cervical carcinoma^11^,^12^. HPV16 E7 also modulates chemokines crucial for the induction of the antiviral CD8 T cell response^43^. Monocyte chemoattractant protein 1 (MCP1), important for infiltration of immune cells to the site of infection, is decreased in the HPV-infected cells^14^, and interleukin 8 (IL-8), a potent chemoattractant for T lymphocytes and neutrophils, is down-regulated by E7^13^.

Depending on their environment, LC antigen presentation can generate activating or tolerising T cell responses^44^. LC have the capacity to migrate to the lymph node and to cross-present skin antigens *in vitro*, activating CD8 T cell responses^20^, and are able to prime CD4 T cells^45^. We predicted that loss of many of the LC in HPV infected skin would reduce the likelihood of LC priming of T cells, thereby contributing to evasion of immunity by the virus. However our work shows that LC are non-essential for activation of the lymph node CD8 T cell response to skin expressed antigen, and that any remaining LC in E7 expressing epidermis are dispensable for the observed suppression of CD8 T cell responses in the lymph node.

The role of LC in the activation of the immune response to skin-expressed antigen remains controversial^46^. Based on *in vitro* data, evidence strongly supports the ability of LC to cross-present antigen to T cells to activate the immune response^47^. More recently, several groups have shown that cross-presentation of skin-expressed antigen can occur in the absence of skin LC^48^. Langerin^+^ CD103^+^ dermal DCs are considered the key cells that cross-present antigens expressed by keratinocytes^48^,^49^. In HPV-infected lesions, dermal DCs are more concentrated immediately beneath the epidermis along the dermo-epidermal junction^50^. The requirement for dermal DCs in presentation of HPV antigens *in vivo* has not yet been demonstrated, however the lack of effect of LC-depletion on T cell proliferation in our study does implicate these cells in presentation of skin-expressed antigen to lymph node T cells. If that is the case, the suppressive effects of E7 are likely to extend to cells that are in the local microenvironment but are not in direct contact with the E7-expressing KC. The density and distribution of the dermal dendritic cells is reduced in patients with persistent HPV-induced anogenital lesions^51^, indicating that dDC may be modulated by HPV. If this is an E7-mediated effect, the loss of dermal DC potentially may contribute to the T cell suppression we observed, and this may impair immunity to HPV.

Collectively, our observations show that expression of the HPV16 E7 protein in KC is sufficient to reduce the number of LC in the epidermis. Although we speculate that LC depletion may contribute to suppression of the immune response to HPV, we provide clear evidence that LC are not required to suppress antigen specific CD8 T cell responses in the skin draining lymph nodes. These data suggest that E7 instead may regulate the dermal cross-presenting APC, the CD103^+^ (mouse) or CLEC9a^+^ (human) langerin positive dermal dendritic cells, which may result in tolerogenic antigen presentation in the draining lymph nodes^52^. The altered activation status of the remaining LCs may have other effects, such as maintenance of skin resident Tregs. It will be critical to determine how antigen presentation occurs in HPV infection, as its regulation may determine viral persistence, the activation of an effective immune response and ultimately viral clearance. The ability to develop therapeutic interventions to trigger regression therefore could hinge on interventions that interfere with regulation by HPV of skin APCs.

## Materials and Methods

### Construction and production of lentiviral vectors

**Figure 5:**
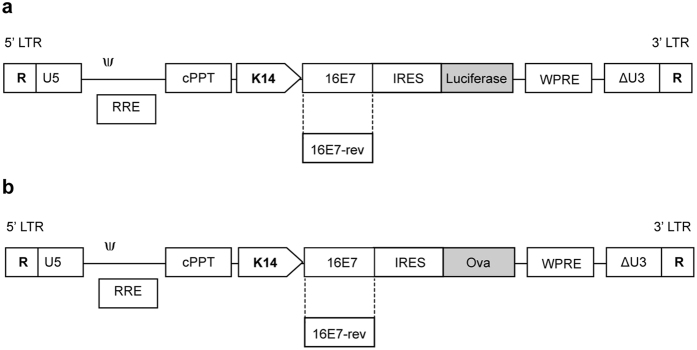
Schematic representation of lentiviral vectors used in the study. Linear maps of the insertion sequences encoding E7 or E7-rev and luciferase (**a**), or ovalbumin (**b**), are shown.

All genetic modifications were approved by the University of Otago Institutional Biosafety Committee and the New Zealand Environmental Protection Agency. Methods were carried out in accordance with the approved guidelines. The lentiviral transfer vector, pRRLSIN-cPPT-PGK-GFP-WPRE (hereafter pRRL-PGK-GFP), was provided by Dr. S. Hughes, Department of Biochemistry, University of Otago and is described in Follenzi *et al*.^53^. pRRL-K14-GFP was generated by replacing the PGK promoter with the 2.1 kb K14 promoter (expressed in basal and suprabasal keratinocytes^54^,^55^) following digestion with *BamH*1 and *Xho*1. The 1.6 kb Luc gene was amplified by PCR from pGL3 luciferase reporter vector (Promega) using the primer pair F-5′-CGCGGATCCATGGAAGACGCCAAAAAC-3′ and R-5′ GCGTGTCGACTTACACGGCGATCTTTCC-3′. pRRL-K14-GFP (linearized with *Bam*H1 and *Sal*1) was ligated with the Luc gene to generate pRRL-K14-Luc. An HPV16 E7-IRES fragment was ligated into pRRL-K14-Luc linearized with *BamH*1 and *Bgl*II, to generate pRRL-K14-E7-IRES-Luc (Fig. 5a). To construct its counterpart negative control, pRRL-K14-E7rev-IRES-Luc, E7 was cloned in the reverse orientation (E7rev). E7rev-IRES was generated by PCR (primer F-5′-GCAGATCTAATACCAAAGACTCTTGT-3′ and R-5′-GCGGATCCTTTTTAACCTCGACTAAA-3′) and ligated into pRRL-K14-Luc (linearized with *BamH*1) to generate pRRL-K14-E7rev-IRES-Luc (Fig. 5a). The 1.1 kb Ova gene was amplified by PCR from pAC-Ova-neo (kind gift from Dr. M. Bevan, University of Washington, Seattle, WA, USA) using F-5′-ATGGATCCATGGGCTCCATCGGCGCA-3′ and R-5′-CGTCGTCGACTTAAGGGGAAACACATCT-3′ and cloned into pRRL-K14-E7-IRES-Luc and K14-E7rev-IRES-Luc, linearized with *BamH*1 and *Sal*1 to generate pRRL-K14-E7-IRES-Ova and pRRL-K14-E7rev-IRES-Ova, respectively (Fig. 5b). Replication deficient lentiviruses were produced following transient co-transfection of 293TT cells with the pRRL-K14 transfer vectors described above, the packaging plasmids pMDLg/pRRE, pRSV Rev and the VSVG plasmid (obtained from Addgene, Cambridge, MA, USA). Supernatant was collected from cells 48 h and 72 h post transfection, filtered through 0.45 μm filter, then centrifuged at 70 000 × *g* for 2 h. Virus titer determination was performed using a real-time quantitative PCR (qPCR) as described in Barde *et al*. (2010).

### Mice and lentivirus transduction

Animal experiments were approved by the University of Otago Animal Ethics Committee (Approval Number: AEC41/10). Methods were carried out in accordance with the approved guidelines. Lang-DTR^56^ and CD45.1 × OT-I (hereafter; CD45.1 OT-I) mice used in this study were bred and maintained under specific pathogen-free conditions in the Hercus Taieri Research Unit (HTRU), University of Otago, New Zealand. Mice were anaesthetised with a mixture of Domitor (1 mg/kg), Ketamine (75 mg/kg) and Atropine (0.05 mg/kg) and revived with Antisedan (1 mg/kg). A total of 1 × 10^7^ lentivirus particles (1:1 mix of 5 × 10^6^ K14 Luc and 5 × 10^6^ K14 Ova lentivirus particles) in 20 μl in PBS were injected intradermally (i.d.) into each ear pinna. In experiments where luciferase expression only was measured, a total volume of 20 μl containing 5 × 10^6^ K14 E7 Luc or K14 E7rev Luc lentivirus particles was injected i.d. into the ear.

### Preparation of epidermal sheets

Ears were split into dorsal and ventral halves, and skin was floated on 3.8% ammonium thiocyanate (ATC, Sigma–Aldrich) in 100 mM sodium phosphate for 20 min at 37 °C. After 20 min, epidermal and dermal sheets were separated using thin curved forceps.

### Western blotting of epidermal sheets

Epidermal sheets were placed in a Dounce homogenizer and disrupted in 50 mM Tris-HCl (pH 7.4), 1% Nonidet P-40, 0.25% sodium deoxycholate, 0.15 M NaCl, 1 mM EGTA and protease inhibitors. Cell lysates were collected and centrifuged at 10,000 × *g* for 5 min. The protein concentration of the sample was determined using a Bradford BCA Quantification Kit (Pierce, Thermo Fisher Scientific) according to manufacturer’s instructions. An equal quantity of protein for each sample was loaded onto a 4–12% polyacrylamide gradient gel (ThermoFisher Scientific, MA, USA), and following electrophoresis samples were blotted onto nitrocellulose using an iBlot Dry Blotting System (ThermoFisher Scientific, MA, USA), as per the manufacturer’s protocol. The membrane was blocked in 5% bovine serum albumin (BSA) and 0.05% Tween-20, in TBS (TTBS) overnight at 4 °C with agitation, then incubated with rabbit anti-Ova antibody (Sigma-Aldrich, MO, USA), or goat anti-actin antibody (C-11, Santa Cruz Biotechnology Inc., TX, USA) diluted in 0.3% BSA in TTBS for 2 h at RT, with agitation. Following further washing, the membrane was incubated with goat anti-rabbit IRDye 800CW or donkey anti-goat IRDye 680RD (LI-COR Biosciences, NE, USA) in 0.3% BSA in TTBS at RT for 60 min. The membrane was washed and then imaged using the Odyssey Clx Imaging System (LI-COR Biosciences, NE, USA).

### Epidermal ear sheet staining and LC enumeration

Epidermal sheets were separated and fixed in acetone for 15 min at RT, transferred to wells of a 24-well plate, washed in 1 ml of TBS containing 0.05% Tween for 10 min, then blocked in 1 ml of TBS containing 1% BSA for 5 min at RT. Sheets were incubated with an Alexa-546 labeled anti-CD207 antibody (Dendritics) diluted in TBS containing 1% BSA for overnight at 4 °C. Sheets were washed twice in TBS for 20 min on a shaking platform at RT, mounted with SlowFade Gold (Invitrogen) on slides and examined using a BX51 (Olympus, France) fluorescence microscope. At least six fields (at 200 fold magnification) within the transduced area were randomly selected and the LC enumerated. All measurements were recorded using ImageJ (http://rsbweb.nih.gov/ij) software.

### Ablation of langerin-expressing cells *in vivo*

LCs were ablated from mouse epidermis by i.p. injection with 1 μg of diphtheria toxin (DT) (Sigma-Aldrich, St Louis, MA, USA) in 100 μl PBS 6 days prior immunization^15^.

### *In vivo* T cell proliferation assay

Cells were harvested from the spleen and lymph nodes of donor CD45.1 x OT-I F1 mice, red blood cells lysed. The remaining cells were labeled with 0.625 μM CFSE (Sigma, MA, USA) in PBS for 8 min at 37 °C, then washed and resuspended in PBS. CFSE-labeled cells (4 × 10^6^) were adoptively transferred intravenously (i.v.) into the tail vain of each recipient mouse seven days following lentiviral vector injection. Five days later, cervical lymph nodes were harvested and single cell suspensions were prepared. The total lymphocyte number was determined using a Z2 Coulter Counter Cell and Particle Counter (Beckman Coulter, CA, USA). Cells were washed, incubated with allophycocyanin (APC)-conjugated anti-CD8a mAb (BD Pharmingen, San Jose, CA, USA) and PerCP-conjugated anti-CD45.1 antibody, washed again and resuspended in 0.5 ml FACS buffer (1% BSA and 0.1% sodium azide in PBS). Propidium iodide (PI; 1 μg/ml) was added to samples prior to analysis by flow cytometry.

### Processing of skin tissue for flow cytometry

Epidermal sheets were transferred into a 50 ml tube containing 20 ml cDMEM and incubated in 37 °C with agitation for 30 min. Cells that were released from the tissue were filtered through a 70 μm cell strainer, centrifuged at 450 × *g* for 5 min at 4 °C, washed and resuspended at 1 × 10^6^ cells/ml in FACS buffer. The cells were incubated with anti-Fc (clone 2.4G2, BD Biosciences) then with APC/Cy7-conjugated anti-CD40 (Biolegend, San Diego, CA, USA), Pacific Blue-conjugated anti-CD80 (Biolegend) and APC-conjugated anti-CD83 antibodies (Biolegend). The cells were fixed and permeabilized using a Fix & Perm kit (Invitrogen, MD, USA), according to manufacturer’s instructions, and incubated with 0.5 μg/ml of PE-conjugated anti-CD207 antibody (Dendritics, Lyon, France). Cells were washed twice and resuspended in FACS buffer prior to flow cytometric analysis.

### Flow cytometric analysis

The cells were analyzed on FACScalibur or FACSfortessa flow cytometers (Becton Dickinson, CA, USA). Lymphocytes were gated on forward scatter (FSC) and side scatter (SSC), and PI positive (dead) cells were excluded from analysis. Unlabelled cells, PI-treated unlabelled cells and single-labelled cells for each fluorescence channel were used to adjust the channel voltages and compensate for the spectral overlap between the fluorochromes. FlowJo version 9.5 (Treestar Inc, CA, USA) was for data analysis.

### Statistical analysis

Comparisons between experimental groups were carried out using a Mann-Whitney U (M-W U) test (Prism 5.0; GraphPad Software, CA, USA). *P* < 0.05 was considered statistically significant.

## Additional Information

**How to cite this article**: Jemon, K. *et al*. Suppression of the CD8 T cell response by human papillomavirus type 16 E7 occurs in Langerhans cell-depleted mice. *Sci. Rep*. **6**, 34789; doi: 10.1038/srep34789 (2016).

## Supplementary Material

Supplementary Information
