# Supplementary material for: Suppression of the CD8 T cell response by human papillomavirus type 16 E7 occurs in Langerhans cell-depleted mice
Source: Sci Rep. 2016 Oct 6;6:34789. doi: 10.1038/srep34789 (PMC5052534; doi:10.1038/srep34789)
Supplement: Supplementary Information [file srep34789-s1.pdf]

Suppression of the CD8 T cell response by human papillomavirus type 16 E7 occurs in Langerhans cell-depleted mice

Jemon, K.[2,3], Leong, C-M.[2], Ly, K[1]., Young, S.L.[1], McLellan, A.D.[2] and *Hibma, M.H.[1]

[1]Department of Pathology, Dunedin School of Medicine, University of Otago, P O Box 56, Dunedin, New Zealand.

[2]Department of Microbiology and Immunology, Otago School of Medical Sciences, University of Otago, Dunedin, New Zealand.

[3]Current address: Faculty of Biosciences and Medical Engineering, Universiti Teknologi Malaysia, Skudai, Johor, Malaysia.

Correspondence to: Merilyn Hibma

Department of Pathology, Dunedin School of Medicine, University of Otago, P O Box 56, Dunedin 9054, New Zealand

Tel: +643 479 7726

E-mail: merilyn.hibma@otago.ac.nz

Keywords: Human papillomavirus, HPV16 E7, Langerhans cells, skin immunity, antigen presentation

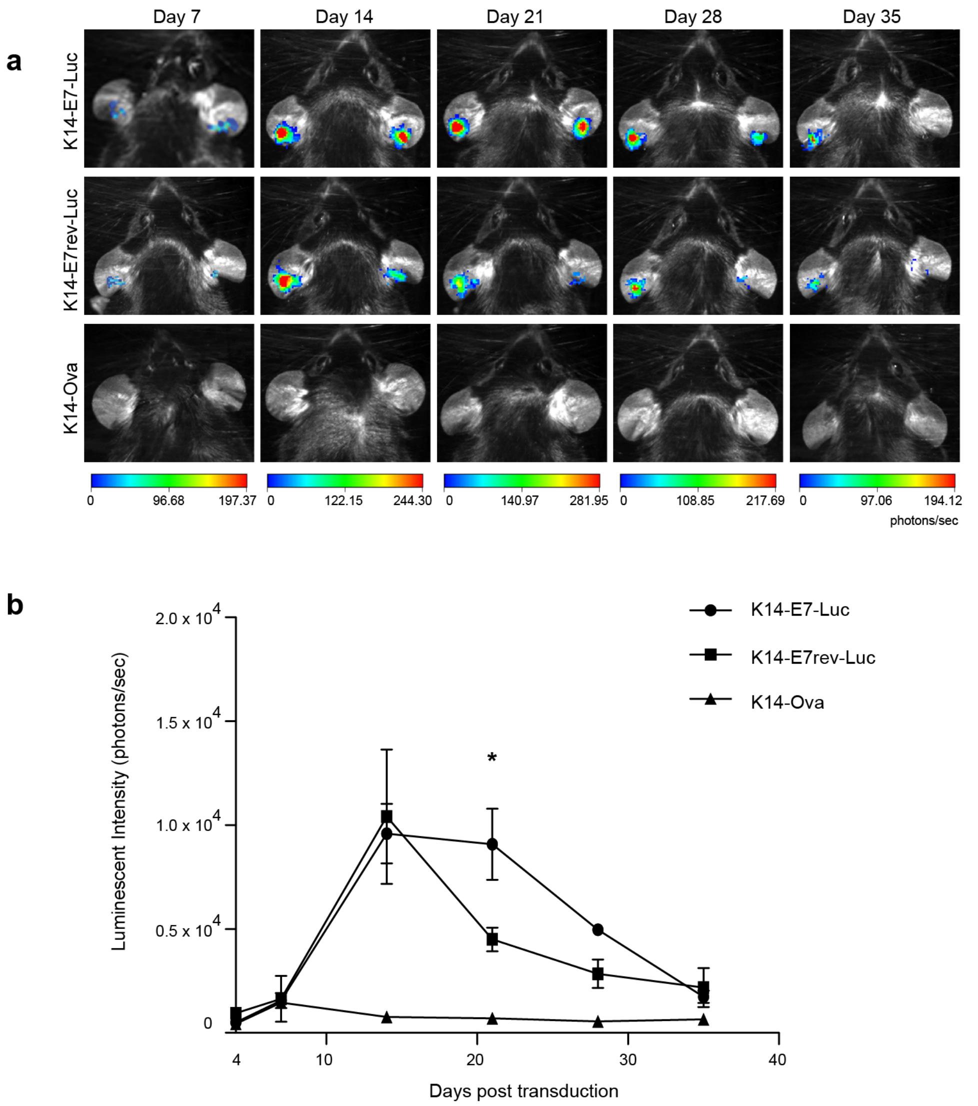

Supplementary Figure 1. Transgene expression persists in K14 E7 expressing mouse skin. (**a**) Lang-DTR mice were injected i.d. with 5×10^6 K14 E7 Luc or K14 E7rev Luc lentivirus particles, or PBS. *In vivo* bioluminescence imaging was carried out over time to measure luciferase gene expression. Expression is indicated by an overlay of the pseudocolour region over the image for each representative mouse. The relative signal intensity (photons/second) is indicated by the colour bars. (**b**) Graphical representation of the luciferase expression in each group (mean ± SEM) n = 4, consisting two ears for each sample in each group; *$P < 0.05$ (Mann-Whitney U), is shown.

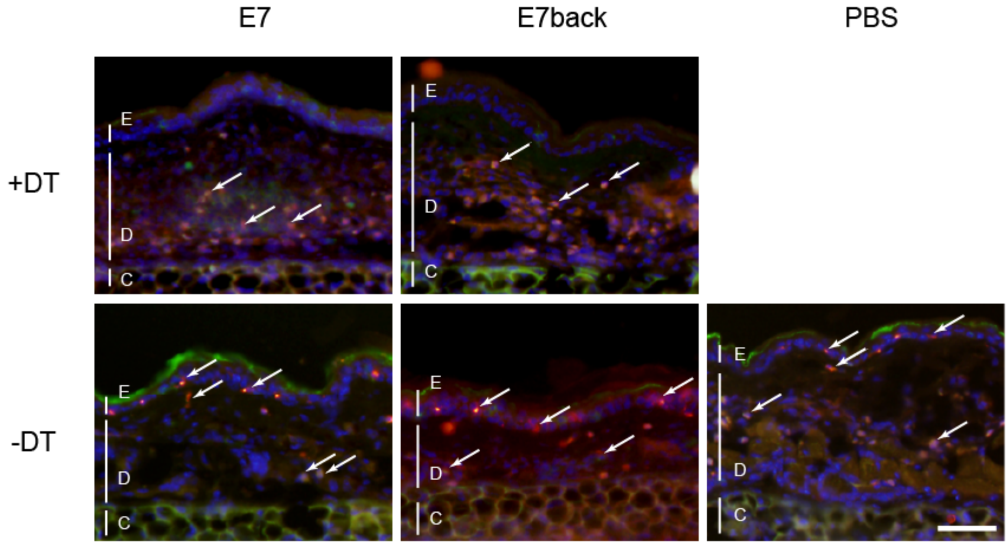

Supplementary Figure 2. LC but not Lang+ dDC remain depleted in ear skin 18 days after DT treatment. Mice were treated with 1 μg of DT i.p. Eighteen days later, tissue sections were stained with antibodies specific for CD207 (red) and CD103 (green). *Upper panel*: Representative images are shown for frozen tissue section of E7 and E7rev transduced mouse ear skin from DT treated mice. *Lower panel*: Representative images are shown for tissue section of mice treated without DT. E, epidermis; D, dermis; C, cartilage. Scale bar, 50 μm. Representative sample from n = 5 mice/group.

Supplementary Methods

In vivo bioluminescent imaging

Mice were injected with 30mg/kg D-luciferin (BioPioneer, San Diego, CA, US) intraperitoneally (i.p.), left for 10 min then imaged with an In Vivo FXPro (Carestream Molecular Imaging, New Heaven, CT, USA) Bioimager for 300 s (100 mm field of view, a binning factor of 8, and an open filter). The region of interest (ROI) was defined manually using a standard ellipse tool, and net signal intensity (sum of the pixels within the band, with the median of the perimeter pixels subtracted) was calculated using Carestream Molecular Imaging software.
